# Supplementary material for: Predation history has no effect on lateralized behavior in Brachyrhaphis rhabdophora
Source: PLoS One. 2023 Feb 15;18(2):e0280900. doi: 10.1371/journal.pone.0280900 (PMC9931090; doi:10.1371/journal.pone.0280900)
Supplement: Attachment — Submitted filename: Response to Reviewers.docx [file pone.0280900.s002.docx]

01 July 2022

To:	PLOS ONE Editor

Subject:	Revision of manuscript/ PONE-D-22-06171

*Predation has no effect on lateralized behavior in* Brachyrhaphis rhabdophora

Dear PLOS ONE Editor,

We have revised our manuscript and resubmit it now after addressing each point raised by you and by the two anonymous reviewers. Our thanks to each of you for taking the time to offer suggestions to improve the paper. These suggestions were very helpful.

We have submitted two versions of our revised manuscript: one with ‘track changes’ turned on so that you can see exactly how we have edited the paper; and one that is a ‘clean version’ with no tracking visible.

As per your instructions, here we provide a ‘rebuttal letter’ detailing each point raised in the initial review. We present this as an itemized list, first presenting the comment from you or from the reviewer (in bold) followed by our response. When we reference line numbers in the bolded comments, these refer to lines in the original manuscript. When we reference line numbers in our response, these refer to the ‘track changes’ version of the revised manuscript.

Finally, all authors have read the revised manuscript and have signed off on the resubmission.

Please contact me if you have any questions.

Maren Callaway

Corresponding Author

**COMMENTS FROM EDITOR**

**1. Please ensure that your manuscript meets PLOS ONE's style requirements, including those for file naming.**

Done.

**2. In your Methods section, please include a comment about the state of the animals following this research. Were they euthanized or housed for use in further research? If any animals were sacrificed by the authors, please include the method of euthanasia and describe any efforts that were undertaken to reduce animal suffering.**

No animals were euthanized in this study. All were returned to the laboratory for potential future use. We have added a sentence at lines 157-158 clarifying this.

**3. We note that the grant information you provided in the ‘Funding Information’ and ‘Financial Disclosure’ sections do not match. When you resubmit, please ensure that you provide the correct grant numbers for the awards you received for your study in the ‘Funding Information’ section.**

Done. Note that the research scholarships from BYU given to authors Maren Callaway and Erik S. Johnson do not have grant identification numbers, so these cannot be included.

**4. Thank you for stating the following in the Acknowledgments Section of your manuscript:  [This research was conducted under the IACUC protocol 18-0803. This work was funded by a CURA scholarships from the BYU College of Life Sciences to MC and ESJ. We thank Javier Guevara Siquiera and Lourdes Vargas Fellas at the Vide Sylvestre, Ministrio del Ambiente y Energia (MINAE), Sistema Nacional de Áreas de Conservación (SINAC), Costa Rica, for processing our collecting permits. Additionally, we appreciate Alli Duffy, Becca White, Trevor Williams, and Megan Pew for contributing to this project by aiding with fieldwork, specimen processing, and fish care.]**

**We note that you have provided funding information that is not currently declared in your Funding Statement. However, funding information should not appear in the Acknowledgments section or other areas of your manuscript. We will only publish funding information present in the Funding Statement section of the online submission form.  Please remove any funding-related text from the manuscript and let us know how you would like to update your Funding Statement. Currently, your Funding Statement reads as follows: [Life Sciences College Underground Research Award (CURA) - MC - Amount awarded: $1500 - URl:** [**https://lscura.byu.edu/**](https://lscura.byu.edu/)**]**

**Please include your amended statements within your cover letter; we will change the online submission form on your behalf.**

We have removed all funding information from the manuscript. The acknowledgement section no longer includes any reference to funding.

As per your request, please update our Funding Statement to read as follows:

“This work was funded by Brigham Young University College of Life Sciences, College Undergraduate Research Awards of $3,000 each to Maren Callaway and Erik S. Johnson.”

**5. Please include your full ethics statement in the ‘Methods’ section of your manuscript file. In your statement, please include the full name of the IRB or ethics committee who approved or waived your study, as well as whether or not you obtained informed written or verbal consent.**

The full ethics statement has been added at lines 107-109 following your guidelines. You will note that we now provide the IACUC protocol number in the revised manuscript.

**6. We note that Figure 1 in your submission contain map/satellite images which may be copyrighted.**

We created Figure 1 using software ArcGIS. It is not copyrighted. We have now appropriately referenced ArcGIS as per the software developer’s guidelines in the legend for Figure 1.

**COMMENTS FROM THE REVIEWERS**

1. Is the manuscript technically sound, and do the data support the conclusions? The manuscript must describe a technically sound piece of scientific research with data that supports the conclusions. Experiments must have been conducted rigorously, with appropriate controls, replication, and sample sizes. The conclusions must be drawn appropriately based on the data presented.

Reviewer #1: No

Reviewer #2: Partly

2. Has the statistical analysis been performed appropriately and rigorously?

Reviewer #1: No

Reviewer #2: Yes

3. Have the authors made all data underlying the findings in their manuscript fully available? The [PLOS Data policy](http://www.plosone.org/static/policies.action#sharing) requires authors to make all data underlying the findings described in their manuscript fully available without restriction, with rare exception (please refer to the Data Availability Statement in the manuscript PDF file). The data should be provided as part of the manuscript or its supporting information, or deposited to a public repository. For example, in addition to summary statistics, the data points behind means, medians and variance measures should be available. If there are restrictions on publicly sharing data—e.g. participant privacy or use of data from a third party—those must be specified.

Reviewer #1: No

Reviewer #2: Yes

4. Is the manuscript presented in an intelligible fashion and written in standard English?

PLOS ONE does not copyedit accepted manuscripts, so the language in submitted articles must be clear, correct, and unambiguous. Any typographical or grammatical errors should be corrected at revision, so please note any specific errors here.

Reviewer #1: Yes

Reviewer #2: Yes

5. Review Comments to the Author

**COMMENTS FROM REVIEWER 1**

1. **This paper set out to determine the impact of predation pressure on laterality in a poeciliid in Costa Rica. Unfortunately, with just 2 rivers examined (1 high and 1 low predation pressure) it can not examine the hypothesis as stated. L75: with just one population from each of high and low predation the authors cannot generalize about the potential impact of predation pressure on laterality. There are presumably any number of environmental variables that differ between these two streams that could result in variation in laterality (or not).**

We appreciate the reviewer asking this question. If we were completely naïve about the *B. rhabdophora* system, we agree that only looking at two sites could present challenges about our inferences. Fortunately, we know quite a lot about *B. rhabdophora* populations based on 25+ years of research in this system. For example, we know that mortality rates differ between these two sites (Johnson & Zuniga-Vega, *Ecology* 2009) and that they are good exemplars of predator and non-predator environments relative to several comparable replicates (Johnson & Belk, *Oecologia* 2001). Also, as we have pointed out in previous work (Johnson & Belk, 2001; Johnson, *Evolution* 2002; Johnson, *Biological Journal of Linnean Soc.* 2002; Ingley et al., *PLOS ONE* 2014), predator environment captures differences in several factors, including the presence or absence of predators, density, canopy cover, etc. But our work has shown that evolutionary divergence in this system is most consistent with differences in mortality rates as the primary selective agent. This is why we have chosen the comparison between these specific two locations—we know that mortality rates differ between these two sites and that several other traits have diverged as a result. The clearest case for this can be found in the 2009 *Ecology* paper references above, which focused on *only* these two locations.

2. **The data about the response to the various stimuli may be useful but it is difficult to know how (if) they should be combined owing to the variation in populations from which they were drawn.**

We are not quite sure if we understand this comment. So, forgive us if we misinterpret the feedback. We chose to consider several different stimuli based on previous research, including work in our own lab that showed that in some species, individuals will show a lateralized detour response to one stimulus type and a different lateralized detour response to a different stimulus type. For example, in our work in the fish *Xenophallus umbratilis*, male fish detour differently in response to predators, mates, and a novel object. We have added a phrase at line 137-138 providing justification and supporting citations for why we chose the stimuli that we did.

3. **The authors also make several statements attempting to enhance the apparent novelty of this study but seem to ignore the literally 100s of papers that have addressed this topic previously. See L35-36.**

We appreciate the reviewer offering this feedback. In response, we have carefully gone through the entire manuscript and removed all statements that could be perceived as trying to oversell the novelty of our work. In many cases, we have replaced these statements with additional information that shows how our work fits into the context of previous studies. It was certainly not our intent to ignore this other important work, and we hope the current version better addresses this literature.

4. **L152 as the opener to the Discussion.**

This appears to be an incomplete thought from the reviewer.

5. **L46: Odd that till now there is no mention of the work on *Brachyrhaphis episcopi*, a sister species where a number of papers have examined high and low predation effects on laterality.**

Thank you for this suggestion. We now introduce the interesting work from Cullum Brown’s lab on *B. episcopi* at line 41, in the second paragraph of the paper. The first paragraph is a more general introduction of laterality. So, this seems like the appropriate place to include it. Good feedback.

6. **L61: I’m not so sure about these hypotheses. If predation drives the evolution and or development of laterality, then shouldn’t the high predation population be highly lateralised irrespective of the stimuli. Otherwise, you are suggesting domain specific laterality which would be rather unusual. Also, these 2 hypotheses are clearly not independent of one another. What we are expecting is a population by stimuli interaction of some sort.**

Thank you for asking us to clarify these hypotheses. Beginning at line 61, we have reworked this penultimate paragraph. We now simply pose our two questions, and then clarify what our expectations are. We think this is more consistent with the suggestion from the reviewer. We don’t intend to imply that our questions (hypotheses) are independent of one another. Indeed, we need to first show that any laterality exists at all before comparing the two populations.

It is perhaps helpful to note that previous work shows that many species display differing lateralized behavior between stimuli (what the reviewer seems to refer to as ‘domain specific laterality’ (see citation 14 and 16, and our response to point #2 above for examples).

7. **L64: I would delete the last line. This is the introduction not the results section.**

Deleted.

8. **L118: did a male have to make a certain number of choices to be included in the data set? Eg more than 10?**

We did not set a minimum threshold in our original analysis. However, upon receiving this feedback, we re-ran our analysis with all trials with fewer than 10 choices excluded. We found that the results did not change. Therefore, we have left the analyses as is. But thank you for this suggestion. There were only a handful of trials with low choice counts, but it is good to know that these are not unduly affecting the results.

9. **L119: it’s important to describe the lighting above the tank and discrepancies in shadow can induce false positives.**

The lighting was the exact same for all trials and was indeed well-lit enough that there is no part of the tank where the fish cannot be seen. We have added a phrase at line 127-129 to clarify.

10. **L139: I’m not sure why there was no attempt to use a 2 way-anova to examine laterality scores. Nor is there any mention of absolute laterality which tends to be a good predictor of the strength of laterality irrespective of direction.**

There are probably a variety of statistical tests that could be used to explore our data. We chose to follow the approach of Torres-Dowdall et al (2019), which ESJ and JBJ have also used (Johnson et al. 2021; see reference 16). By using a laterality score and testing for a deviation from zero, we are also able to test if our results differ from random, and if so by what magnitude.

11. **Fig 1? My version doesn’t seem to have a map.**

We suggest following up with the editorial office to find out how to access the map in the review files.

12. **Fig 3. Why 2 SE?**

Two standard errors have the helpful property of framing the 95% confidence interval of the true mean. Hence, for readers who know this, they can simply check if the CI’s overlap to quickly ask if the mean values are statistically different from each other.

13. **L161-167: This explanation does not make sense. Clearly populations within a species vary with respect to predation regime, so to suggest that a sister species ought to show the same pattern due to a common ancestor is irrational. Moreover, Bisazza and colleagues have conducted multiple comparisons across species and found that schooling tendency predicted laterality not phylogeny.**

Thank you for articulating your discontent with this part of the discussion. In response, we have completely reworked these first two paragraphs. Beginning at line 213 we offer a more focused explanation for why laterality might simply not exist in our focal species. We do still mention phylogenetic history but do so as a way to possibly frame future work. We invite the reviewers to read through this and see if it makes more sense.

14. **L168: also not true. There are multiple papers examining the costs and benefits of laterality in poeciliids as well as in vertebrates more generally, including multiple reviews.**

We have deleted this statement and replaced it with a more accurate statement about what we know of costs and benefits of laterality in poeciliid fishes. It now reads: “We know that in general there can be benefits and costs of laterality.” We invite additional feedback on our revised discussion to see if it aligns with the knowledge and expertise of the reviewer.

16. **L178: There is most certainly a publication bias. I have three students working on laterality in 5 species and is not reliably showing up in any of them.**

Thank you for sharing this. It does beg the larger question about why some species so reliably show laterality and others do not. We look forward to seeing more of your work come out on this topic.

**COMMENTS FROM REVIEWER 2**

Glad to read this paper that aimed at investigating behavioural lateralisation in a poeciliid fish depending on predation history. Although well written and organized, my opinion is that there are some flawless in the current form of the paper.

1. **First of all it is not clear to me how many trials were recorded in order to compute the index of laterality for each subject during the 20min of observation, whether or not they have discarded subjects that does not spontaneously turn or if they tend make "proper turn" toward one or the other end of the apparatus.**

Please see our response to point #8 for Reviewer #1 above. In most trials, each fish made a choice between 20 and 50 times over a 20-minute period. In other words, there was a lot of data for each trial. Moreover, the apparatus that we used has previously been demonstrated to be effective in detecting laterality and it follows a standard design used by multiple research labs. We have confidence that we have collected good data, consistent with the norm in this field of research.

2. **I was also wondering about the possibility to test for possible behavioural asymmetries using a viewing situation in which the subject is required to make closer inspection to the stimuli.**

This is an interesting idea. We used the detour test here for a couple of reasons. First, it is an established method and has been widely used in the realm of lateralization work. Second, by using a standard approach, it allows us to compare our work to other published studies. We have confidence that this approach is valid. But as the reviewer suggests, understanding decision making with more visual information at closer distances might offer additional insights. It’s just not something that we did in this study.

3. **Finally why do not include females?**

Please see our explanation at lines 118-122. We intentionally chose males so that we could compare our work with previous work. Also, males are more likely than females to be motivated to seek out potential mates. Hence, using males allowed us to explore the effects of potential mates more effectively; or at least that was our rationale with the design of the study.

5. **In the discussion section (line 167) the authors claim that there are "too few tests of laterality....", not sure what they are arguing here but actually theres a quite large literature on how to assess lateralization in poeciliid fish. In general the result section appear to be pretty much concise.**

Thank you for this suggestion. We have removed this phrase. In fact, at your suggestion and that from the other reviewer, we have modified the manuscript extensively to avoid overselling the novelty of our work.

6. **Minor comments: reference 31: Giorgio V. should be Vallortigara G**

We have corrected this. We have also carefully reviewed all of our citations to look for additional mistakes. Thanks for you input here.
