# Supplementary material for: Predation history has no effect on lateralized behavior in Brachyrhaphis rhabdophora
Source: PLoS One. 2023 Feb 15;18(2):e0280900. doi: 10.1371/journal.pone.0280900 (PMC9931090; doi:10.1371/journal.pone.0280900)
Supplement: Attachment — Submitted filename: Response to Reviewers 2.docx [file pone.0280900.s003.docx]

22 October 2022

Dear Editor,

Thank you for the additional input on our manuscript titled “*Predation history has no effect on lateralized behavior in* Brachyrhaphis rhabdophora*” (*PONE-D-22-06171R1). We have carefully read the comments made by you and by the two reviewers, and we have revised our manuscript accordingly. Here we provide an itemized list of each point raised by the reviewers (listed in bold text), along with our response (listed in non-bold text).

We appreciate this additional input. It has furthered improved the paper, and we hope that you will find it acceptable for publication. Please contact me if there are any further edits that you would like us to make. We look forward to hearing from you.

Sincerely,

Maren Callaway

**REVIEWER #1 – Professor Culum Brown**
This paper has certainly improved, and I thank the authors for taking on and addressing previous concerns. I confess, even given previous work on this system, I’m still uncomfortable with n = 1. Having done many of these projects myself, I recognize how much work goes into it, and I want to be sure the authors have really explored their data as best they can. With this in mind, I’ve offered some alternative analyses that they might consider. The methods still require a bit more detail so folks could replicate them.

**1. Line 65 - I still don’t feel comfortable with the one population from high and one from low predation environments. These populations undoubtedly differ in any number of ways which may influence laterality. However, given the historical context (Johnson and Belk 2001) it is reasonably likely that the variation may be due to predation. I would be far happier to see multiple pops tested.**

We appreciate this comment. Unfortunately, we do not have replicate populations in the laboratory to conduct additional tests. However, we have done a couple of things in the revised manuscript that we hope will acknowledge the concern and still allow us draw conclusions from the study. First, we have added language to the revised manuscript that makes clear that when we refer to ‘predation environment’ in the paper, that we include not only the presence or absence of predators, but also other ecological components associated with these environment types. Specifically, we refer to Johnson (2002) which included a very transparent description of these habitat types. This should make it clear to the reader that predator environments include several factors, which is certainly the case for previous work done in *B. episcopi* and guppies. It’s not an ideal response, but it does acknowledge previous work in similar systems. Second, we also refer to published work (Johnson and Zuniga-Vega 2009) showing that these two specific populations are known to differ in mortality rates. We don’t know exactly all the factors that contribute to these differences—although the cichlid fish predator must be an important part of this—but we do know that for the two localities evaluated here, there are measured differences. Combined, we hope this will at least add some confidence to the comparison between the two sites chosen. It’s not perfect, but it is also not as fraught with confounders in other systems where less is known.

**2. L126: It’s also not clear how long males recovered before being tested again. (inter-trial period). Also state here that you did not set a minimum number of turns for the males to qualify and perhaps make a statement about how many runs the fish did make.**

Thank you for asking for this clarification. We agree, it is important to make sure that there are not some trials with very few turns that that are unduly influencing our results. Consequently, we re-ran our analyses including only trials where fish made at least five turns—these results also showed no differences among populations. In essence, nothing changed. That said, we did clarify this in our revised manuscript in the Methods section (line 111). We also now report how long the resting period was between trials (line 124).

**3. L144: I’m still a bit confused by the statistics. I can appreciate that the current method tells us if the estimates of LI overlap with 0, but I still think its worth-while exploring if they differ with pop and treatment as this speaks directly to your hypotheses. In this case, would it not be better to include a mixed model with individual ID as the random variable, pop and treatment as independent variables and LI as the dependent?**

This is a sound suggestion, especially as it allows us to compare our results directly to the work previously done with *B. episcopi*. As recommended, we ran a mixed model repeated measures ANOVA (with all samples included), treating *population* and *treatment* as independent variables, and *individual* as a random variable (see lines 153-155). Consistent with our previous analyses of LI for each treatment, we found no differences between predator environments, and no differences among our treatments.

**4. L157: I would be interested to know if any of the individuals were lateralised in their response to various cues. What does the distribution look like? Where strongly lateralised fish consistently strongly lateralised? Previous papers, for instance, have examined the proportion of the population tested that scored at 70% L or R (lateralised). I just get the feeling you could do more with this data and individual details get lost in population averages.**

Thanks for this suggestion. We went back to our data and found that none of the individuals in our study had LI values that exceeded positive or negative 0.7. We report this in our Results (line 162).

**5. L203: Of course, there are other explanations, including low sample sizes or inappropriate experimental design, which are less interesting but nonetheless significant concerns.**

Our sample size and methodological approach follow typical conventions for this type of study. Using a similar approach, Johnson et al. (2020) found evidence for lateralized behavior in *Xenophallus umbratilis*, and Brown et al (2004) found evidence for lateralized behavior in *B. episcopi* from predator environments. Hence, we feel reasonably confident that our findings are real, and not just due to design issues.

**REVIEWER #3**

The paper of Callaway and colleagues investigates the effect of predation history on eye preference in the livebearing fish *Brachyrhaphis rhabdophora* by detour test. The authors reported no evidence for behavioral lateralization at all in response to any stimuli (predator, mate, novel, control), regardless of predation population or predation-free population.

This paper is written clearly and the unique behavioral experiment has been conducted. Appropriate corrections have already been noted by the reviewers. I feel that this manuscript provides a novel contribution to the field. Interesting concept and idea but there is a lot of variation in the behavioral data and no explanation for this. Also, I point out that there are problems with the experimental design. Please make a couple of improvements as follows:
Introduction

**6. P4, line 48-53: It is necessary to explain in detail how much mortality in the fish differs between environments with and without predators.**

Thank you for this request. One of the strengths of this system is that we know mortality rates by size class for *B. rhabdophora* from these two environments. This work is published in Johnson and Zuniga-Vega 2009. We have included a brief reference to these mortality rates in our Methods section where we describe the study system.

**7. Methods. P7, line127-129: Insufficient information about the experimental procedure: in what order were the four stimuli presented? Were the stimuli random order? The interval of the experiment was short (10 minutes), and it is suspected that the subjects may become accustomed to the experimental test and feel tired.**

Thank you, these are all important questions that we have now addressed in the methods of our paper. In brief, we ran the control treatment first and the predator treatment last for each species, with the other two treatments in between. We have added an explanation for this order at line 122 of the revised paper. As mentioned above, we explored the possibility that fish might have become accustomed to the test and therefore decreased their activity and detour behavior. We addressed this by examining if fish conducted fewer detours in the latter half of a trial; we found no evidence for this.

**8. Results. Figure 3 shows that there is a very large variation in the data, and we must consider the causes of this variation. This is because this result is directly related to the conclusion that there is no laterality in the livebearing fish. One possibility is that a detour test of 20 minutes may be too long for observation. Even at the beginning and end of the experiment, the laterality tendency eye may change (Cantalupo et al. Neuropsychologia 1995, Sovrano et al. Physiology and Behavior 2001, Bisazza et al. Behavioural Brain Research 2002). If LI is calculated for only the first 5 or 10 minutes, will the results change? Longer experiment time introduces the effect of habituation and learning, which makes the interpretation of results more difficult.**

This was a good suggestion to evaluate. We re-ran our analyses including only the first 10 minutes of data in each trial. We found no difference in our results. Habituation does not appear to be an issue here.

**9. The number of trials per individual is not specified in the results. It is unfortunate that this has not been corrected in the text, even though this was pointed out by Reviewer 2.**

As stated on line 122, each individual participated in four trials, one for each stimulus.

**10. Discussion. P9, line167-171: It is necessary to specify what type of left-right behavior is exhibited by the three closely related species of this species. Whether there is a unifying trend or not would allow us to consider the need to question the phylogenetic background.**

Of the four species that we refer to here, three are distantly related and one is closely related. We do not have information on behavioral lateralization in all species in the family. What we know is this. The closely-related species *B. episcopi* shows behavioral lateralization in individuals that occur with predators, but not in individuals that occur with no predators. The other three species are from distantly related genera. Hence, it seems unlikely that we are dealing with an issue of phylogenetic history governing laterality patterns. Though there is evidence of heritability of laterality in some species (line 178), we know laterality and phylogenetic history are independent of each other in another fish family, Belontidae (line 184). As such, we question the phylogenetic background given there is evidence for both possibilities.

**11. P10, line181-183: "non-lateralized individuals tend to choose higher-quality shoals than lateralized individuals". What is meant by “higher-quality shoals” and how it is costly needs to be explained.**

Thank you for asking for this clarification. We have added a phrase in this sentence (line 193) that defines what we mean by high-quality shoals: shoals with more individuals and shoals with individuals of roughly the same size as the fish present. By costly, we simply refer to the idea that the larger the shoal the greater the dilution for each individual. We also note that individuals of the same size are less conspicuous than individuals of different sizes.
